# Observations on the Crural Hernia

**Published:** 1804-01-01

**Authors:** John Hull

**Affiliations:** Manchester


### *To the Editors of the Medical and Physical Journal.*

Gentlemen,

THE attention of the profession having been particularly called of late to the subject of crural hernia, by two very valuable publications, viz. Mr. Hey's Practical Observations in Surgery, and Dr. Monro's, (jun.) Observaons on Crural Hernia, I am induced to send you some Remarks relating to this disease; and will thank you to give them a place in the next Number of your useful Journal. I am, &c.

*Manchester, Nov.* 20, 1803. JOHN HULL.

---

### Observations on the Crural Hernia.

The Crural Hernia, or Merocele, is generally considered as a protrusion, or descent of a portion of the contents of the abdomen, included in a sac derived from an elongation of of the peritonæum, under the crural arch. By a few authors of high reputation, this genus of hernia has been allotted a more extensive signification, being made to comprehend other ruptures, that appear in the bend of the thigh, in which the protruded viscus passes *over* the crural arch, through a separation of the fibres of the transverse and internal oblique muscles and of the aponeurosis of the external oblique muscle of the abdomen. Chopart and Dessault,* as far as I have been able to determine, are the first writers who have noticed the latter kind of hernia; and it has since been admitted as a species of crural hernia by Richter† and Callisen.‡ None of the above mentioned authors have given a detailed account or case of this hernia as passing over the crural arch, so that it is not easy to determine whether it be a conjectural or real species. If such a hernia have been observed, I should think it may with more propriety be referred to another genus; to the inguinal hernia, if it accompany the spermatic vessels, or round ligament of the womb, in their egress from the abdomen; and to the ventral hernia, if the protrusion take place in a point where no vessels are passing out of the abdomen. I shall therefore take no further notice of it in this paper.

The true crural hernia is supposed by Garengeot to have been known to Paul and Barbette. Sabatier also considers the latter as acquainted with it. However this may be, its existence appears to me to have been first properly established by Verheyen, professor of Anatomy at Louvain, in the year 1694, from the dissection of a soldier, who died in the hospital of that city from an incarcerated merocele. The particular circumstances of this case may be seen in Palfyn's Anatomie Chirurg. P. ii. p. 73. Ed. 3.

A considerable diversity of opinion has prevailed among Anatomists and Surgeons with respect to, 1st. the relative situation of the crural hernia to the crural vessels; 2dly,

---

* La hernie située au pli de la cuisse se nomme crurale; les viscères passent derrière le ligament de Fallope, &c.—On sortent au-dessus de ce ligament par un écartement des fibres des muscles transverse, oblique interne & de l'aponeurose de l'oblique externe. *Traité des Maladies Chirurg.* Tom. 2, p. 205.

† "Zuweilen liegt dieser bruch gar nicht unter dem Poupartschen bande, sondern er tritt durch eine spalte in den muskelfibern über dem Poupartschen bande." *Abhandlung von den Brüchen,* p. 650.

‡ "Hernia cruralis sub margine ligamenti Fallopii in spatio, vasis iliacis decurrentibus dicato, interdum supra ligamentum Fallopii per diductas fibras aponeuroticas, exoritur." *Syst. Chir. Hodiernæ,* T. ii. sect. 748.

the part which produces the stricture, when it is incarcerated; and, 3dly, the proper method of removing the stricture by a chirurgical operation. The remarks which I have to offer at present, relate principally to these three circumstances. I think it necessary to premise a concise description of the crural arch, and to notice the course of the different blood-vessels liable to be wounded by the division of the arch, because a good deal of confusion and some apparent contradictions are met with in the writings upon this subject, for want of precision in the employment of terms.

The crural arch, which, from the real or supposed discoverer of it, has been named the *ligament of Fallopius* or *Poupart;* from its situation and texture the *inguinal and crural ligament,* the *doubled tendon of the external oblique muscle, &c.* is rather irregular in its form and low in proportion to its extent. Its external extremity or foot, is attached to the superior and anterior spinous process of the os ilium; its internal extremity to the spinous process and part of the crista of the os pubis; hence it is considerably higher at its external than its internal extremity. By many anatomists this arch is stated to be tendinous, and is considered as formed by the duplicature of the lower aponeurotic border of the external oblique muscle only. By others it is considered as a distinct ligament, to which the lower border of the external oblique muscle is attached. This dispute about the tendinous or ligamentous nature of the arch may, I apprehend, be settled by the following observation made by Soemerring. "Certe etiam tendines nil sunt, nisi ligamenta musculorum." *De Corporis Humani Fabrica.*" Tom. iii. sect. 73.

The crural arch has a groove, or canal, along its upper surface, which is deeper nearer to its insertion into the os pubis, and is formed by the tendon, or ligament, being doubled backwards and upwards. The anterior margin of the arch, or that which is turned towards the integuments, is thicker and rounded; the posterior margin, which is turned towards the peritonæum, is thinner and sharper to the feel.

Under the crural arch are situated the external iliac and great psoas muscles, the anterior crural nerves, the external iliac blood-vessels, and the lymphatics of the lower extremity.

Nearly in the middle of the arch the external iliac artery passes to the thigh, and there takes the name of the crural artery. The iliac vein is situated a little more inwards than the artery. The space between the iliac artery and and the outer extremity of the arch is completely filled by the iliacus internus and psoas magnus muscles and the anterior crural nerves, so that in the natural state of the parts no aperture is met with there. But that part of the arch, which is situated more inwards than the iliac blood-vessels, is not equally filled, a small aperture being always found between the iliac vein and the internal foot of the arch. This aperture, to which Gimbernat has very properly given the name of the *crural ring*, is larger in the female than in the male. It is bounded above by the crural arch and below by the os pubis. Towards the abdomen it is covered only by the peritoneum; hence a portion of omentum, intestine, &c. may pass through this aperture to the thigh, and carrying the peritoneum along with it, may form the crural hernia. And we can imitate this hernia pretty well after opening the abdomen, by passing a finger from thence through the crural ring to the thigh. Below, or towards the thigh, this ring is covered by the fascia lata, so that the hernial sac cannot be brought into view till the fascia has been divided. The fascia lata being intimately connected with the crural arch, gives it a considerable degree of tension when the thigh is extended, and relaxes it considerably when the thigh is bent to a right angle with the trunk. The posterior margin of the crural arch, which has been mentioned above as feeling sharp to the finger, is much less distinct when the thigh is bent; a circumstance necessary to be attended to, when we employ the taxis, as it then presents less resistance to the return of the prolapsed viscus.

For an external view of the crural arch, the reader is referred to Mr. Ch. Bell's System of Dissections, plates 13 and 14, and to Dr. Monro's (jun.) plate 5, fig. 1. A good internal view of the crural arch and ring is given by Dr. Monro, (jun.) in plate 3, fig. 1, as it appears in the female subject; and in plate 4, fig. 2, as it appears in the male. It was the more necessary to give figures of these parts in both sexes, as the Doctor has discovered a material difference in the male and female, the crural ring being smaller and the posterior part of the arch being much broader in the former, especially at and near its insertion into the os pubis.

The epigastric artery arises from the external iliac, a little before the latter passes under the crural arch. It in general first passes down a little way, and then passing obliquely upwards and inwards along the outer and upper part of the crural ring, continues its course behind the in-

 ternal ternal oblique and transverse muscles, but before the peritoneum, till it reaches the posterior side of the rectus abdominis. Sometimes, however, it passes immediately upwards and inwards, thus keeping at a greater distance from the crural ring. See Monro's Plates, 6 and 5

The spermatic artery in the male, in its course from the abdomen to the testicle, first passes on the outside of the epigastric artery, that is, between it and the iliac artery, and then crosses the epigastric most commonly directly over the crural ring. It passes out of the abdomen under the inferior margin of the transverse muscle, and through the lower margin of the internal oblique. The spermatic artery and vein being joined by the vas deferens, and furnished with a muscular covering, named the cremaster, chiefly from the internal oblique, form the spermatic cord, which passes downwards for about two inches between the internal and external oblique muscles in the groove on the upper part of the crural arch, before it arrives at the inguinal ring. Hence it appears, that the spermatic vessels are much more superficially seated than the epigastric artery, after they have crossed it, and may be exposed by an incision made through the lower part of the external oblique only, just above the crural arch. This is a circumstance deserving particular attention.

The round ligament of the womb, in the female, passes out of the abdomen, and crosses the epigastric artery in the same manner as the spermatic vessels do in the male; it afterwards descends in the same way along the canal in the upper part of the crural arch to the ring of the external oblique muscle. See Monro's plates, 6 and 5.

When a hernia is formed in the male by the descent of a portion of one or more of the abdominal viscera through the crural ring, the epigastric artery passes along the outer and upper side of the neck of the sac, and is liable to be wounded by an incision made upwards and outwards thro' the crural ring; the spermatic artery passes along the upper and inner side of the neck of the sac, and is liable to be divided if the ring be cut upwards and inwards; and both these arteries, when they cross each other directly over the neck of the sac, (which is most commonly the case) are liable to be wounded by cutting through the crural ring directly upwards. These arteries sometimes cross each other a little on the outside of the sac, in which case the spermatic cord only will be divided by cutting through the crural ring directly upwards, the epigastric artery being situated beyond the reach of the knife.

In

In the female the above observations will apply, substituting the round ligament of the womb for the spermatic vessels. See Monro's plates, 6 and 5.

The division of the epigastric artery in the operation for the crural hernia, has, in various instances, occasioned a fatal hæmorrhage. And as it has always been found extremely difficult to tie this artery, we should take great care to avoid wounding it.

The division of the spermatic cord will also produce a dangerous hæmorrhage; and, though this should be restrained, the testicle of that side of the body may be rendered useless by the division of the spermatic artery or the vas deferens. It is therefore of great consequence that the spermatic cord should not be wounded in the operation for the crural hernia.

The division of both the epigastric artery and the spermatic cord, which may happen when the crural arch is cut through directly under the point where these vessels cross each other, must necessarily prove still more embarrassing to the operator.

In operating upon the female, the epigastric artery is principally deserving of attention, because the division of the round ligament of the womb will not be attended with any very serious consequences, especially in women beyond the period of child-bearing.

In the usual distribution of the blood-vessels, the arteria obturatoria arises from the iliaca interna, or some of its branches, and passes to the foramen thyroideum, quite out of the reach of the knife; but, in some cases, this artery is so situated as to be liable to be wounded in dividing the crural arch: for it sometimes arises from the iliaca externa by one common trunk with the epigastric artery; and when this trunk is long, the arteria obturatoria has been found passing first along the upper and then on the inside of the neck of the hernial sac, so as almost to surround it. Mr. Thompson, of Edinburgh, has observed this course of the artery in six out of ten preparations which he had examined, and considers it as an insuperable objection to the mode of operating in crural hernia proposed by Gimbernat, (see Monro, jun. p. 66.) When the common trunk of the arteria epigastrica and obturatoria is short, the latter is observed passing down on the outside of the neck of the sac, so as not to be in danger of being wounded. See Monro's plate 4, f. 1.

### § 1. *Of the Situation of the Crural Hernia, with respect to the Crural Blood-vessels.*

Several authors of eminence, among whom we may mention Chopart and Dessault, Richter, Mr. B. Bell, and Herin, are of opinion that the crural hernia, though more commonly placed on the inside of the crural vessels, is sometimes situated immediately before them, and even on the outside of them, or nearer the iliac extremity of the crural arch.

Gimbernat, on the other hand, contends, that this hernia is always situated on the inside of the crural vessels, or nearer the symphysis pubis; and Dr. Monro, jun. seems to agree with him.

I have not met with any case recorded, in which the crural hernia was originally situated on the outside of the crural artery; and though I would not venture to deny the possibility of such an occurrence, yet I should not expect to find this hernia in any other situation than on the inside of the crural vessels, (protruded through the crural ring), when it first takes place, or whilst it continues of small size. In all the cases of crural hernia, which have fallen under my observation, the size of the tumor was from that of a hazel nut to that of a walnut, except in one, which I attended some years since with Mr. Nanfan. In this instance the tumor was nearly as large as the head of a new born child, and occupied almost the whole of the superior and anterior part of the thigh. It had been many years in an irreducible state, and when I was called in, the integuments covering the tumor were highly inflamed, and a gangrene had taken place in the centre, to a considerable extent. The hernial sac was not laid open by the separation of the slough, and the old man, after some time, got well. He is since dead, and I regret much that the opportunity of examining this hernia by dissection was lost. As but few cases are recorded, wherein the crural hernia has attained a large size, it may not be improper to state here, that Le Dran has related a case, in which the hernial sac was eight inches in circumference, and three inches in depth; and that M. de la Faye, in his Notes to Dionis's *Cours d' Operations de Chirurgie*, p. 339. ed. 4. Paris 1740, has asserted, that this hernia has been known to extend to the middle of the thigh. In these rare cases, where the hernia, from its bulk, is necessarily found situated before and on the outside, as well as on the inside, of the crural vessels, I think it highly probable, that the descent descent of bowel first took place through the crural ring on the inside of the large blood-vessels, and that the ring had gradually yielded on the outside, so as to allow the hernia to extend laterally in that direction. We know that the inguinal ring has, in some instances, been so far dilated as to allow three fingers, or more, to pass through it with ease; and Le Dran found, in the case mentioned above, that the Fallopian ligament had yielded so much, that one might almost thrust four fingers, with the integuments, underneath it. *Obs. de Chirurg.* T. ii. Observ. 58. If this opinion be correct, it will also follow, that the epigastric artery, and spermatic cord, or round ligament of the womb, according to the sex of the patient, will be carried outwards with the hernia, and that the epigastric artery will be still situated on the outside of the neck of the sac, and liable to be wounded by making the incision of the arch upwards and outwards.

### § 2. *Of the Situation of the Stricture in the incarcerated Crural Hernia.*

It is unquestionably of the greatest importance to an operator, to know in what different ways and situations the stricture may be formed in crural hernia, otherwise he may be liable to much embarrassment. He may also be led to divide a part unnecessarily, or may leave the part undivided, which really produces the stricture. In the latter case it is obvious that the operation will prove of no advantage to the patient.

Gimbernat tells us, that it is the thin posterior border of the crural arch "which always forms the strangulation."

Many writers consider the rounded anterior margin of the crural arch, as being frequently the seat of the stricture.

By Günz, Bertrandi, Richter, * Callisen, and Arneman, it is stated, that the stricture is sometimes produced by the fibres passing from the fascia lata of the thigh into the crural arch.

Mr. Hey, of Leeds, has asserted very lately, that the stricture is not caused by Poupart's ligament, but by another part, situated three-eighths of an inch below Pou-

* Es scheint, dass zuweilen bloss die flechsenfasern, die ans der fascia lata ins Poupartsche bandgehen, die ursache der einklemmung sind; wenigstens hat man geschen, (Günz, Bertrandi) dass sich der bruch so gleich Zurück bringen liess, als diese fasern durchschnitten waren. *Anfangsgründe der Wundarzneykunst.* B. 5. § 530.

part's ligament, and which, by way of distinction, he has named the *femoral* ligament.

It has been long ascertained, that the stricture, in crural hernia, may be occasioned by the hard and contracted state of the neck of the sac. Le Dran, in the year 1726, performed the operation for this hernia in a male, and found the strangulation occasioned by the neck of the sac. "He says, "Je m'apperçus aussi-tôt après, que ce n'étoit pas le ligament, qui faisoit le plus fort étranglement & que c'étoit l'entrée du sac, qui, ayant été long-temps comprimée par la pelotte du brayer, s'étoit retrecie. Alors y portant mon doigt, je sentis, que ce sac ressembloit à une bourse fermée & que son entrée seule étoit capable d'empêcher la reduction des parties, tant elle étoit étroite." *Observ. de Chir.* Tome ii. p. 6. He also opened the body of another man, in the same year, and found the strangulation occasioned by the neck of the sac, which, with its contents, had been returned into the abdomen. *Ibid.* p. 14. And Dr. Monro has mentioned a case, that occurred to him in the year 1782, in which a small portion of the ilium was strangulated by a straitness and thickening of the neck of the sac. *Description of all the Bursæ Mucosæ, &c.* p. 49.

And Mr. Cline, in his valuable lectures on Hernia, states, that septa, or partitions, are occasionally found in the hernial sac below its neck, by which a stricture may be induced; and shews a preparation of a bubonocele, with three of these septa formed within the sac. I do not know that any of these septa have hitherto been observed in the sac of a crural hernia; but they may be regarded as a possible cause of strangulation in this genus of hernia.

§ 3. *Of the means proposed and employed for removing the Stricture in incarcerated Crural Hernia.*

The division of the fascia lata, where it lies over the hernial sac, has, in some instances, enabled the operator to return the protruded parts, without cutting or dilating the crural arch. It may therefore be worth while to try this method before we undertake to divide the arch; but in general, I believe, it will prove insufficient. The manner of dividing the neck of the hernial sac, when this is the seat of the stricture, has been very ably pointed out by Dr. Monro, sen. and will be considered hereafter, when I come to deliver my opinion on the best method of performing forming the operation, under the different circumstances that may occur.

The means proposed for removing the stricture produced by the crural arch, which is by far the most frequent cause of strangulation in the hernia under consideration, will now be examined. These may be referred to two heads, viz. the *dilatation* and the *incision* of the arch.

1st. In consequence of the danger arising from wounding the epigastric artery and spermatic cord in cutting the crural arch, the mere dilatation of it has been recommended, and two instruments have been invented for the purpose; a curved levator, or blunt hook, by M. Arnaud, and a dilator, with two branches, somewhat in the form of a gorget, by M. Le Blanc. The attempt to dilate the crural arch, is disapproved of by Mr. B. Bell, and by Gimbernat; but recommended by Chopart and Dessault, Richter, Callisen and Arneman. Richter, indeed, is of opinion, that a surgeon is not justified in cutting the crural arch, before he has tried to dilate it by Arnaud's hook, or Le Blanc's dilator.

I have not tried either of these instruments; and though I should not expect much advantage from the use of them, I do not see any particular objection to making an attempt to dilate the crural arch, provided care be taken not to injure the subjacent parts. The hook appears to me to be the preferable instrument, as it affects the arch only; whilst the dilator can scarcely be managed, so as not to injure the protruded parts, or crural vessels.

2d. With respect to the *division* of the crural arch, authors in general are agreed, that the *extent* of it should be no greater than is necessary to allow the return of the protruded viscus, or viscera. They differ, however, very much as to the *part* of the arch, which ought to be divided, the *direction* in which the division ought to be made, and the *manner* of executing it.

In giving an account of the opinions of the different systematic and practical writers upon this subject, it may be attended with some advantage to refer them to the six following classes.

The 1st, comprehending those writers who have not distinctly pointed out the particular direction in which the division of the arch ought to be made, as Garengeot, Heister, Pott, &c.

The 2d, comprehending those who direct the incision to be made upwards and outwards, as Sharp.

The

The 3d, comprehending those who direct the incision to be made upwards and inwards; *e. g.* Le Dran, &c.

The 4th, comprehending those who direct the incision to be made straight across, or directly upwards, as Dr. Monro, sen. &c.

The 5th, comprehending those who direct the incision to be made directly inwards, where the posterior margin of the arch is inserted into the os pubis, as Gimbernat and Latta.

The 6th, comprehending those who are of opinion, that the division of the arch is to be made in different directions, according as the hernia is differently situated, with respect to the crural vessels, as Chopart and Dessault, Richter, Lassus, &c.

Upon the whole, however, it will be found more convenient to give their opinions, as nearly as I well can, in a chronological order; and this will be done as concisely as possible.

Garengeot is the first author, whom I have hitherto met with, that has published cases of the operation for the crural hernia. He informs us, that he had seen the wound of a woman dressed in May 1718, whom Mr. Petit had operated upon with success, without opening the hernial sac; and he has related two cases, which occurred to himself in the years 1720 and 1728. In the former of these, a winged director was passed under the crural arch, and the bistory was scarcely introduced into the groove of the director, before the arch was relaxed, and the intestine returned spontaneously.—*Traité des Opérations de Chirurgie.* Tome i. p. 28. Ed. 2. Paris 1731. 12mo. *Memoires de l'Acad. Roy. de Chir.* Tome ii. p. 457. 12mo.

2. Heister is still less explicit than Garengeot. He merely says, "Ampliari paululum foramen illud, unde intestina proruperunt, oportet.' *Inst. Chir.* Pars 2. p. 820. Amstelad. 1739, 4to.

3. Le Dran directs us to cut the crural arch, by carrying the incision obliquely towards the linea alba, and to make it very small, because the epigastric artery is near.. *Traité des Oper. de Chir.* Paris 1742.

4. Platner gives no direction whatever for the division of the crural arch.—*Inst. Chir. Rat.* § 849, 8vo. Lips. 1745.

5. Sharp, both in his Treatise on the Operations of Surgery, and in his Critical Inquiry, directs the crural arch to be cut obliquely outwards, as the spermatic vessels will thus be avoided. He allows that in this way the epigastric artery may be divided; but he thinks this accident ought ought not to embarrrass an operator, as it may *instantly* be taken up by the needle.

6. Pott states, that if the incision of the ligament be made of any length, let it be made in whatever part it may, the risque will be great of wounding either the spermatic cord, or the epigastric artery. He seems to depend too much on our being able to return the contents of the hernial sac without dividing the crural arch, "as there is a considerable space between the os ilium and the os pubis, to manage such reduction in." He says, it is not so easy to take up the epigastric artery as to direct it to be done; but observes, that the spermatic cord is certainly more to be regarded than it. He merely directs us, if the division of the crural arch be unavoidable, to be particularly careful to keep the extremity of the probe-pointed knife within the end of the fore-finger, and to make as small an incision as may be necessary.—*Chirurgical Works,* vol. 2, p. 153, Lond. 1783, 8vo.

It would appear that Pott did not write from observation, in directing the point of the knife to be kept within the end of the finger, and that he was not very well acquainted with the anatomy of the crural arch, from his declaration, relative to the space for managing the reduction in,

[To be continued.]

